# PDE12 in type 1 diabetes

**DOI:** 10.1038/s41598-022-22890-x

**Published:** 2022-10-28

**Authors:** Hasim Tekin, Knud Josefsen, Lars Krogvold, Knut Dahl-Jørgensen, Ivan Gerling, Flemming Pociot, Karsten Buschard

**Affiliations:** 1 The Bartholin Institute, Department of Pathology, Rigshospitalet, Copenhagen Biocenter, Ole Maaløes Vej 5, 2200 Copenhagen N, Denmark; 2 Division of Paediatric and Adolescent Medicine, Oslo University Hospital, Oslo, Norway; 3 Department of Medicine, University of Tennessee, Memphis, TN USA; 4 Steno Diabetes Center Copenhagen, Gentofte, Denmark; 5 Faculty of Health and Medical Sciences, University of Copenhagen, Copenhagen, Denmark; 6 Faculty of Medicine, University of Oslo, Oslo, Norway

**Keywords:** Medical research, Pathogenesis, Diabetes, Type 1 diabetes, Viral infection

## Abstract

Type 1 diabetes (T1D) incidence is increased after COVID-19 infection in children under 18 years of age. Interferon-α-activated oligoadenylate synthetase and downstream RNAseL activation degrade pathogen RNA, but can also damage host RNA when RNAseL activity is poorly regulated. One such regulator is PDE12 which degrades 2′-5′ oligoadenylate units, thereby decreasing RNAseL activity. We analyzed *PDE12* expression in islets from non-diabetic donors, individuals with newly (median disease duration 35 days) and recently (5 years) diagnosed T1D, and individuals with type 2 diabetes (T2D). We also analyzed *PDE12* single-nucleotide polymorphisms (SNPs) relative to T1D incidence. *PDE12* expression was decreased in individuals with recently diagnosed T1D, in three of five individuals with newly diagnosed T1D, but not in individuals with T2D. Two rare *PDE12* SNPs were found to have odds ratios of 1.80 and 1.74 for T1D development. We discuss whether decreased *PDE12* expression after COVID-19 infection might be part of the up to 2.5-fold increase in T1D incidence.

## Introduction

Recent research has shown that the incidence of type 1 diabetes (T1D) is increased up to 2.5-fold after coronavirus disease 2019 (COVID-19) infection in children under 18 years of age^1,2^. Similar increases in new-onset T1D have also been reported in adults^3^. One theory that explains how viral infections may lead to T1D involves the interferon (IFN)-α-activated latent ribonuclease (RNAseL) signaling pathway^4^. When IFN-α mediated cell stimulation induces downstream activation of 2′-5′ oligoadenylate synthetases (OASs), the high levels of 2*′*-5′ oligoadenylate (2-5A) produced bind to and activate RNaseL. Excessive RNaseL activity may lead to the degradation of both pathogen and host RNA, thereby causing cellular damage^5,6^. This activity is regulated by phosphodiesterases such as PDE12, which degrade 2-5A molecules, suppressing RNaseL activation. In fact, a direct link between PDE12 and OAS has been described in a PDE12-null HeLa cell line^7^. PDE12-null cells were also resistant to infection with encephalomyocarditis virus, human rhinovirus and respiratory syncytial virus, highlighting a protective effect that is associated with decreased PDE12 activity and thereby increased RNaseL activity. In addition, a separate study on inflammatory pathways in patients with T1D found that PDE12 levels are decreased in the peripheral blood of individuals with new-onset T1D (i.e., mean diabetes duration of 0.22 years)^8^.

## Results

**Figure 1:**
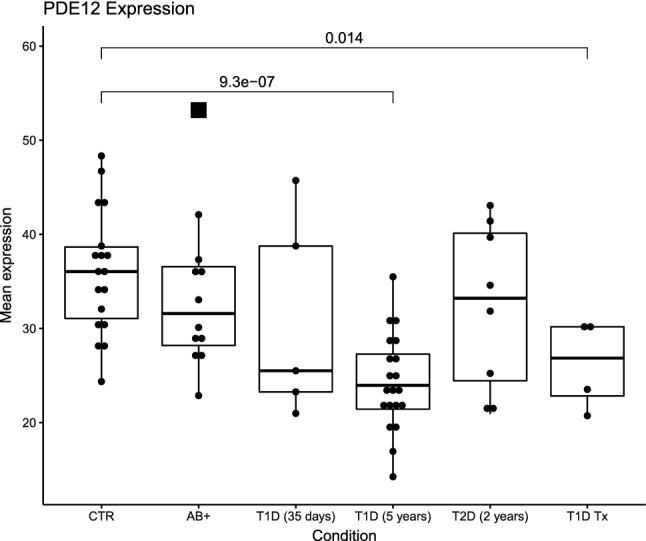
Phosphodiesterase 12 (*PDE12*) gene expression. CTR: non-diabetic controls (*n* = 18); AB + : non-diabetic autoimmune antibody-positive donors (*n* = 12); T1D (median disease duration, 35 days): donors with newly diagnosed type 1 diabetes (*n* = 5); T1D (median 5 years): donors with recently diagnosed type 1 diabetes (*n* = 20); T2D (median 2 years): donors with type 2 diabetes (*n* = 8); T1D Tx: biopsies from donors with recurrent T1D (*n* = 4). Boxes indicate 25% and 75% quartiles, whiskers 1.5 × interquartile ranges, and squares mark outliers. The *p*-values shown were calculated using unpaired two-sided *t*-tests relative to CTR. Test statistics for CTR vs T1D (5 years): t-statistic 6.054, 95%CI 7.74;15.59, degrees of freedom 31.997, mean of CTR 35.95, mean of T1D (5 years) 24.29. Test statistics for CTR vs T1D Tx: t-statistic 3.43, degrees of freedom 5.87, 95%CI 2.78;16.81, mean of T1D Tx 26.16.

From the Affymetrix analysis (Fig. 1), we observed significant decreases in *PDE12* expression for the islets of individuals with recently diagnosed T1D (median disease duration, 5.0 years) and for islets from biopsies originating from donors with recurrent T1D after pancreas transplantation. *PDE12* expression was also decreased in autoantibody-positive individuals, but not significantly. Furthermore, three of the five individuals with newly diagnosed T1D (median disease duration, 35 days) exhibited low levels of *PDE12* expression. However, *PDE12* expression was not altered in individuals with type 2 diabetes (median disease duration, 2.0 years) (Table 1).

**Table 1:** Demographics and clinical status of the pancreas donors used in the Affymetrix analysis.

Clinical diagnosis	Age	Biological Sex	BMI (kg/m2)	Duration of diabetes (years)	C-peptide (nmol/L)	Hb1Ac (%)	Peak glucose (mg/dL)
No diabetes	65	Male	24.2	0	2.8	0	212
No diabetes	21	Male	27.8	0	3.52	0	0
No diabetes	30	Male	20.6	0	17.91	0	279
No diabetes	16	Male	14.9	0	2.94	0	211
No diabetes	68	Female	23.7	0	2.97	0	208
No diabetes	14.2	Male	30	0	5.37	0	249
No diabetes	38	Male	21.7	0	11.1	6	183
No diabetes	22.7	Male	28.9	0	7.61	0	312
No diabetes	51	Male	25.2	0	0.00	6.2	336
No diabetes	17	Female	26.4	0	2.75	0	1039
No diabetes	42.9	Female	23.4	0	0.51	5.2	0
No diabetes	45.8	Female	25	0	4.45	5.6	256
No diabetes	45.1	Female	35.1	0	0.55	6.1	292
No diabetes	31	Female	26.9	0	6.23	5.5	221
No diabetes	33	Female	29.5	0	1.92	5.3	153
No diabetes	47	Female	19.7	0	0.00	0	177
No diabetes	21.8	Female	20.7	0	2.74	0	167
No diabetes	42	Male	31	0	0.47	5.6	298
T1D	22.6	Female	21.6	7	< 0.05	0	494
T1D	14.2	Male	26.3	4	< 0.05	0	425
T1D	31.2	Male	27	5	< 0.05	0	526
T1D	27.1	Male	25.9	11	< 0.05	0	363
T1D	21	Female	22.8	1.5	< 0.05	0	1499
T1D	13	Male	21.3	5	0.42	13.1	645
T1D	13	Male	17.4	0	0.1	13.3	664
T1D	5	Female	11.95	0.25	0.1	0	587
T1D	37.2	Female	30.9	20	0.2	0	630
T1D	18.8	Female	25.2	8	< 0.05	0	1105
T1D	22.9	Male	28.8	7	0.00	0	256
T1D	19.2	Male	23.7	5	< 0.05	0	509
T1D	12	Male	20.3	1	0.18	0	480
T1D	12	Female	26.6	3	0.05	9.8	310
T1D	11	Male	12.9	8	0.06	0	824
T1D	26	Female	26.6	15	0.48	0	860
T1D	24	Female	24.4	4	< 0.05	10.5	615
T1D	13.1	Female	24.8	1.58	< 0.05	0	248
T1D	12	Female	22	9	< 0.05	8.9	641
T1D	43.5	Male	28.7	21	< 0.05	0	0
AB +	69.2	Female	21.3	0	1.84	0	226
AB +	23.2	Female	17.6	0	2.01	5.4	267
AB +	40.3	Male	29.7	0	0.51	5.6	449
AB +	37	Male	26.3	0	5.43	0	185
AB +	4.3	Female	14.8	0	8.95	0	342
AB +	41.4	Male	27.4	0	13.55	0	0
AB +	64.8	Male	34.3	0	26.18	0	0
AB +	48.5	Female	24.5	0	< 0.05	0	440
AB +	40	Male	19.8	0	13.34	0	259
AB +	31.9	Male	21.9	0	0.06	0	196
AB +	22	Male	28.2	0	17.48	5.5	160
AB +	23.8	Female	32.9	0	3.19	5.2	287
T2D	36.1	Male	30.6	0	3.45	7.2	332
T2D	42.8	Male	31	2	0.58	7.8	400
T2D	45	Female	32.3	15	4.17	0	209
T2D	48	Male	41	2	3.46	0	247
T2D	45	Female	39.1	2	3.17	0	286
T2D	62	Female	19.9	10	6.14	6	265
T2D	18.8	Female	39.3	0.25	10.68	0	373
T2D	20.7	Female	40	0	0.58	0	553
Newly diagnosed T1D	35	Male	26.7	0.096	N/A	7.1	N/A
Newly diagnosed T1D	24	Female	28.6	0.096	N/A	7.4	N/A
Newly diagnosed T1D	31	Male	25.6	0.096	N/A	7.4	N/A
Newly diagnosed T1D	34	Female	23.7	0.173	N/A	7.1	N/A
Newly diagnosed T1D	24	Male	20.9	0.057	N/A	10.3	N/A
T1D Tx	49	Male	23.1	0	N/A	N/A	N/A
T1D Tx	40	Male	22.7	0	N/A	N/A	N/A
T1D Tx	38	Female	24.7	0	N/A	N/A	N/A
T1D Tx	63	Male	26	0	N/A	N/A	N/A

“T1D” describes donors with recent (median disease duration, 5.0 years) disease, “Newly diagnosed T1D” donors which were diagnosed with a median of 35 days prior to pancreas donation, “T1D Tx” biopsies from donors with recurrent T1D, “AB + ” autoantibody-positive but not clinically diagnosed donors, “T2D” donors with T2D. “Peak glucose” is the highest measurement taken at the hospital. “N/A” indicates that the given attribute has not been measured.

The single-nucleotide polymorphism (SNP) analysis revealed that individuals with the two rare *PDE12* SNP variants shown in Table 2 had an odds ratio of 1.80 and 1.74 for developing T1D.

**Table 2:** SNPs close to *PDE12* that were associated with type 1 diabetes.

Position	Allele	MAF	dbSNP	p-value	OR	Consequence
3:57.547.247	T/C	0.001	rs143375472	1.77e−6	1.80	3ʹ-UTR variant
3:57.562.439	G/T	0.0005	rs536228505	0.00053	1.74	Intron variant

SNPs close to the *PDE12* gene that were associated with the development of T1D were identified at two positions within the human genome. Abbreviations: PDE12, phosphodiesterase 12; MAF, minor allele frequency; SNP, single-nucleotide polymorphism; T1D, type 1 diabetes; UTR, untranslated region.

## Discussion

The observed decrease in *PDE12* expression seems to have a protective effect against viral infections because it upregulates RNaseL activity in beta cells and other cells^7^; however, it may have the unfortunate side effect of triggering beta-cell damage and subsequent diabetes pathogenesis. Vaccines against COVID-19 should not activate the RNaseL cascade and therefore should not increase the incidence of T1D. Prolonged RNaseL activity may damage and kill cells^9^. Therefore, RNaseL activity must be carefully regulated to protect against viruses without compromising cellular function. Consequently, any treatments that inhibit PDE12 activity and thereby stimulate antiviral defenses should only be given for short durations, to prevent damage to cells. In fact, we found that *PDE12* expression levels are decreased in individuals with recently diagnosed T1D (median disease duration, 5.0 years). During viral infection, which may initiate T1D development, individuals have high levels of PDE12 activity which makes combating the virus difficult. Then, in the post-virus phase there is a decrease in *PDE12* expression which leads to beta-cell damage. Here, stimulating *PDE12* expression might have inhibited T1D development.

The link between COVID-19 and T1D supports the theory that viruses can act as pathogenic triggers for T1D^1,3^. Recent research has shown that severe acute respiratory syndrome coronavirus 2 (SARS‑CoV‑2) decreases insulin expression and induces transdifferentiation of beta cells from COVID-19-infected and deceased donors^10,11^. Furthermore, beta cells readily express the angiotensin converting enzyme 2 (ACE2) receptor^12^ used by SARS-CoV-2 for host entry, and βTC3 cells and isolated rat beta cells show substantially higher 2-5A activity upon IFN-α stimulation when compared to αTC3 cells or rat alpha cells^13^. These observations may explain why beta cells are at increased risk of RNaseL-mediated cellular damage upon viral challenge, even though the virus itself is not toxic. Together, these data might support the increased incidence of T1D after COVID-19 infection and provide valuable insight into the pathogenesis of T1D. However, several other mechanisms for the comorbidity has been suggested including the ACE2-receptor and pro-inflammatory cytokine changes^14^. Since our study is fairly small, it is not possible at this point to have a firm conclusion of the relationship between COVID-19 and T1D. However, the PDE12 hypothesis seems not to be in conflict with the other mechanisms just mentioned.

## Methods

### Human tissue

Pancreatic tissue from donors was collected in the Diabetes Virus Detection (DiViD)^15^ and Network for Pancreatic Organ Donors with Diabetes (nPOD)^16^ studies, with informed consent obtained from all participants. Briefly, DiViD donors with diabetes had a surgical resection of the pancreatic tail, between three and nine weeks after their type 1 diabetes diagnosis, while nPOD material originates from cadaveric organ donors (see Table 1). The procedures were approved by The Norwegian Government’s Regional Ethics Committee (reference 2009/1907); nPOD donors with approval by the University of Tennessee Health Science Center (UTHSC) local Institutional Review Board (reference 10–00848-XM). All experiments were performed in accordance with relevant guidelines and regulations.

### Microdissection of pancreatic islets

Acquired pancreatic samples were laser microdissected as described previously^17^. Briefly, frozen tissue sections from nPOD and DiViD was microdissected with the Arcturus Pixcell II laser capture microdissection system (Arcturus Bioscience, Mountain View, CA, USA). Islets from 2 to 5 sections per donor were detected by autofluorescence and pooled together, and afterwards subjected to RNA extraction with the Arcturus PicoPure RNA Isolation Kit (Applied Biosystems, Grand Island, NY, USA). RNA quality and quantity was validated with the Bioanalyzer 2100 (Agilent Technologies, Santa Clara, CA, USA), and samples underwent gene expression analysis with the Affymetrix expression arrays (Thermo Fisher, Santa Clara, CA, USA) as described previously^18^.

### SNP analysis

Genotyping data were retrieved from the UCSD T1D GWAS meta-analysis^19^ which includes samples from 501,638 control individuals and 18,942 patients with T1D. Similarly, the T2D multi-ethnic meta-analysis^20^ includes samples from nearly 1.2 million control subjects and 228,499 T2D cases.

### Statistics

*PDE12* expression statistics were calculated using Welch’s *t*-test and visualized with R software (ver. 4.1.2; R Development Core Team, 2021) using the tidyverse (ver. 1.3.1), ggplot2 (ver. 3.3.5), and ggpubr (ver. 0.4.0) packages.

### Ethical approval

DiViD and nPOD studies were approved by The Norwegian government’s regional ethics committee (reference 2009/1907) and by the University of Tennessee Health Science Center’s local institutional review board (reference 10-00848-XM).

## Data Availability

Data have been deposited with datadryad.org https://doi.org/10.5061/dryad.d7wm37q4b. The protocols used can be obtained upon request to the corresponding author. Researchers interested in acquiring biological sample from the donors can apply through the DiViD and nPOD programs.

## Code availability

The code used to produce visuals and statistics for Fig. 1 can be obtained upon request from the corresponding author.
